# Analysis of water consumption behavior and savings of Isparta people

**DOI:** 10.1038/s41598-026-51330-3

**Published:** 2026-05-23

**Authors:** M. Erol Keskin, Tevfik Aslanbaş

**Affiliations:** 1 Department of Civil Engineering, Süleyman Demirel University, Isparta, Turkey; 2 State Hydraulic Works (DSI), Isparta, Turkey

**Keywords:** Water conservation, Use and consumption Pearson correlation analysis, Statistical method, Engineering, Environmental sciences, Environmental social sciences

## Abstract

Water is an indispensable resource essential for sustaining life on Earth. Sustainable access to safe drinking and utility water is essential for human survival. Considering the potential of water on the earth and the rapid increase in the population, saving the existing potential water gains importance. The behavior of consumers in saving water has the same level of importance all over the world. The tendency of the public regarding the way of water use and consumption can be found by various methods. In order to determine this tendency in Isparta city center, a survey study was conducted on the subject. The subjects were randomly selected in Isparta. Participants were asked 23 questions to determine their water use and consumption patterns, and statistical methods were used to analyze the answers obtained from the questionnaire. The results obtained are presented in tables and graphs.

## Introduction

Water is one of the most important elements that play a major role in the continuation of life in the world. Consumers’ behavior in saving water has the same level of importance all over the world. The tendency of the public regarding the way they use and consume water can be found by various methods. It is known that.

Chen et al.^1^ demonstrated that social capital plays a significant role in water-saving behavior, showing that households with higher levels of social capital tend to conserve more water than those with lower levels. Similarly, Larson and Tveit^2^ examined urban water demand management strategies and reported that these approaches can effectively support water conservation and the sustainable use of water resources. Stringer et al.^3^ emphasized that improved demand and supply management, supported by effective infrastructure and governance, contributes to more equitable access to water resources and enhances overall water security. Basu et al.^4^ highlighted that a lack of community ownership can limit participation in the operation and maintenance of water systems, while also pointing to an ongoing shift toward quality infrastructure, regular maintenance, and proactive monitoring in rural water management. Casas et al.^5^ found that demographic and behavioral factors such as age distribution, household size, and environmental attitudes significantly influence water consumption, whereas climatic and urban density variables may not always be significant.

Almulhim and Abubakar^6^ stressed the importance of understanding residential water consumption patterns for designing effective behavioral interventions, identifying key socio-demographic determinants such as education, income, household size, and housing characteristics. In a similar vein, Grespan et al.^7^ reported that both socio-economic and structural housing characteristics, including building type and the presence of water-intensive amenities, significantly affect household water use. Koop et al.^8^ reviewed existing studies and concluded that research on domestic water conservation has largely focused on socio-economic drivers and behavioral intentions, while Araya et al.^9^ emphasized the importance of incorporating household-specific characteristics into water consumption analyses. Furthermore, Singha et al.^10^ showed that positive attitudes, awareness, and participation in conservation initiatives significantly increase the likelihood of adopting water-saving behaviors. Finally, Stoker and Rothfeder^11^ analyzed the influence of climatic, demographic, and environmental variables on urban water use, demonstrating that these factors may vary depending on land use patterns.

In Isparta province with a population of 200.000, water consumption and usage patterns of users affecting water consumption were statistically analyzed.

## Material and method

This study employs a cross-sectional survey design to examine household water use and water conservation behaviors in the city center of Isparta. The primary objective is to identify statistical relationships among self-reported water use practices, rather than to establish causal effects or predictive models.

The dataset for the study was collected through face-to-face surveys conducted with 455 households. These households were selected from 30 neighborhoods in the city center of Isparta with populations exceeding 2000 between 2009 and 2013, and the subscriber information was obtained from at least 15 households in each neighborhood where the water subscription had been held by the same person for at least four years. The sample was selected using a systematic sampling method.

All methods were carried out in accordance with relevant guidelines and regulations. The questionnaire was designed to ensure anonymity and confidentiality of participants.

Selecting households that had had the same water account holder for at least four years ensured consistency in reported water usage practices. To maintain urban comparability, only neighborhoods with a population of over 2000 were included. While the sample does not claim to fully represent the entire city population, it provides a sufficiently broad and diverse dataset for an exploratory statistical analysis of household-level water usage behaviors.

A total of 455 surveys were administered to the sample groups. The surveys were completed through face-to-face interviews with households. Since the dataset contained more than 30 observations, the applicability of parametric methods was first tested. The results of the statistical analyses indicated that the continuous data followed a normal distribution. Participants’ water usage and consumption patterns were examined. The distribution characteristics of the data were assessed using the Kolmogorov–Smirnov test and supported by measures of skewness and kurtosis. Therefore, Pearson correlation analysis was applied to examine the linear relationships between variables.

The survey instrument consists of 23 items measured using a five-point Likert scale ranging from 1 (“strongly disagree”) to 5 (“strongly agree”). Higher scores indicate more frequent or consistent participation in water-saving behaviors. In line with the descriptive scope of the study, the scale focuses on observable behaviors rather than attitudes or perceptions. The analysis focuses on behavioral patterns rather than attitudes or perceptions.

This study adopts a cross-sectional survey design to examine self-reported household water use and conservation practices. The methodological focus is descriptive and exploratory, aiming to identify relationships among reported behaviors rather than causal relationships or predictive models. The chosen approach is suitable for providing an empirical overview of household water usage trends within a specific urban context.

### One sample Kolmogorov–Smirnov compliance test

The Kolmogorov- Smirnov goodness-of-fit test in a Group is used to determine how well a random sample fits a particular distribution (flat, normal, or Poisson). Using this test, one can determine whether a series is normally distributed^12^: 132. That is, it investigates whether the distribution of sample values conforms to a predetermined theoretical distribution^13^: 260,^14^: 158. The null hypothesis is "observed frequencies match the expected frequencies". This test is an alternative to the χ2 conformity test. For the χ2 test to be applicable, each expected frequency must be at least equal to 5. However, the Kolmogorov–Smirnov test does not impose a lower limit for the expected frequency^15^: 153.

### Pearson correlation analysis

Co-variance is defined as an increase, decrease or inverse relationship between two variables, regardless of which of the two variables is independent and which is dependent. In statistics, the measure of co-change between variables is revealed by the correlation coefficient^16^. In Eq. (1), ${r}_{xy}$ is the Pearson correlation coefficient and it varies between − 1 ≤ ${r}_{xy}$  ≤  + 1. When ${r}_{xy}$ is − 1, there is a negative correlation between X and Y variables, and when ${r}_{xy}$ is + 1, there is a positive correlation between X and Y variables. A value of 0 for ${r}_{xy}$ indicates that there is no relationship between the two variables^17^.

1 $$r_{xy} = \frac{{\sum\limits_{i = 1}^{n} {(X_{i} - \overline{X})} (Y_{i} - \overline{Y})}}{{\left[ {\sum\limits_{i = 1}^{n} {(X_{i} - \overline{X})^{2} } \cdot \sum\limits_{i = 1}^{n} {(Y_{i} - \overline{Y})^{2} } } \right]^{1/2} }} = \frac{{S_{xy} }}{{\sqrt {S_{xx} S_{yy} } }}$$

The Pearson correlation coefficient is defined to measure the strength and direction of the linear relationship between two variables. The variables used in the equation are defined as follows:

- ${r}_{xy}$: Pearson correlation coefficient between variables *X* and *Y*
- *X*: First variable (e.g., a specific water use behavior)
- *Y*: Second variable (e.g., another water use behavior)
- $\overline{X}$: Mean value of variable *X*
- $\overline{Y}$: Mean value of variable *Y*
- *n*: Number of observations

If the number of variables is more than two and normally distributed, the partial correlation coefficient is calculated from Eq. (2).

2 $$r_{xy.z} = \frac{{r_{xy} - r_{xz} r_{yz} }}{{\sqrt {(1 - r_{xz}^{2} )(1 - r_{yz}^{2} )} }}$$

In Eq. (2), xy z r . denotes the partial correlation coefficient between variables X and Y when variable Z is held constant.

The variables in the equation are defined as follows:

- ${r}_{xy\cdot z}$: Partial correlation coefficient between variables *X* and *Y*, controlling for *Z*
- ${r}_{xy}$: Pearson correlation coefficient between *X* and *Y*
- ${r}_{xz}$: Pearson correlation coefficient between *X* and *Z*
- ${r}_{yz}$: Pearson correlation coefficient between *Y* and *Z*

This formulation allows the isolation of the direct relationship between *X* and *Y* by removing the influence of variable *Z*.

Pearson correlation analysis was selected due to the approximately normal distribution of most items and the study’s aim of examining linear associations between continuous Likert-scale variables. Given the exploratory nature of the research, correlation analysis was considered appropriate to identify co-occurrence patterns among water use behaviors rather than to establish causal relationships or predictive effects.

### Ethics approval and consent to participate

In this study, all procedures involving human participants were conducted in accordance with the ethical standards of the institutional and/or national research ethics committee and the 1964 Declaration of Helsinki and its subsequent amendments.

The study protocol and all data used in this study have been reviewed and approved by the (SDU Institute of Natural Sciences.)

Informed consent was obtained from all participants included in the study. Participation was voluntary, and participants were informed about the purpose of the study and their right to withdraw at any time without any consequences.

## Results and discussion

### Data analysis

The data set of the study was larger than 30, so the One Sample Kolmogorov–Smirnov method given in Table 1 below was used to check whether the data were normally distributed. Since Sig. values were less than 0.05 with this method, there was no normal distribution. The histogram does not show a normal distribution. The normality test with the One Sample Kolmogorov–Smirnov method is more widely and validly used in Basic Sciences, and in other fields, the Skewness and Kurtosis values of the data given in Table 2 below are checked. If the Skewness and Kurtosis values are between − 1.5 and + 1.5, the data are normally distributed^18^. Again, if Skewness and Kurtosis values are between − 2 and + 2, the data are normally distributed.^19^. Skewness and Kurtosis values of the majority of the data set in the study are between − 1.5 and + 1.5. Since very few of them are between − 2 and + 2, the data set is normally distributed.

**Table 1:** Normal distribution test of the data set by one sample Kolmogorov–Smirnov method.

Water use consumption patterns	Kolmogorov–Smirnov		
	Statistics	df	Sig
[ 1) I keep my shower time short.]	0.228	224.00	0.00
[ 2) I use low-flow shower heads]	0.361	224.00	0.00
[ 3) When showering and bathing, I do not run the water continuously, I turn the faucet on and off.]	0.180	224.00	0.00
[ 4) I take my bath in a bucket of water.]	0.216	224.00	0.00
[ 5) Instead of flushing, I pour water down the toilet with a bucket]	0.219	224.00	0.00
[ 6) I use the saving siphon with a small and a large tank inside the body.]	0.351	224.00	0.00
[ 7) I start the washing machine when it’s full.]	0.360	224.00	0.00
[ 8) I choose short programs in the washing machine]	0.200	224.00	0.00
[ 9) When buying a new washing machine, I make sure it has a water-saving feature.]	0.260	224.00	0.00
[ 10) I turn off the tap when shaving or brushing my teeth (for men).]	0.216	224.00	0.00
[ 11) I leave the faucet running during general cleaning]	0.289	224.00	0.00
[ 12) I don’t wash the dishes by hand, instead I wash them in the machine to save money.]	0.223	224.00	0.00
[ 13) When I wash the dishes by hand, I wash them in a bowl, not under a running tap ]	0.238	224.00	0.00
[ 14) I wash the dishes when the machine is full.]	0.228	224.00	0.00
[ 15) When buying a new dishwasher, I make sure that it has a water-saving feature]	0.248	224.00	0.00
[ 16) I wash fruit and vegetables in the container to save money]	0.281	224.00	0.00
[ 17)I wipe the balconies clean instead of washing them.]	0.360	224.00	0.00
[ 18)I check the plumbing for leaks (e.g. dripping faucet, leaking toilet) I fix the leak immediately.]	0.230	224.00	0.00
[ 19)When buying a water-powered household appliance, I pay attention to its water saving feature..]	0.235	224.00	0.00
[ 20) I report any water leaks I come across to the authorities.]	0.340	224.00	0.00
[ 21) I make sure to use a broom instead of a hose when cleaning in front of garage doors.]	0.364	224.00	0.00
[ 22) I use a bucket rather than a hose when washing the car.]	0.363	224.00	0.00
[ 23) I avoid watering the garden during the hottest hours of the day.]	0.378	224.00	0.00

**Table 2:** Skewness and kurtosis values of the data set.

Water use consumption patterns	Skewness	Kurtosis
[ 1) I keep my shower time short.]	0.273	− 1.390
[ 2) I use low-flow shower heads]	0.834	− 0.873
[ 3) When showering and bathing, I do not run the water continuously, I turn the faucet on and off.]	− 0.282	− 1.316
[ 4) I take my bath in a bucket of water.]	− 0.136	− 1.562
[ 5) Instead of flushing, I pour water down the toilet with a bucket]	− 0.387	− 1.420
[ 6) I use the saving siphon with a small and a large tank inside the body.]	1.145	0.008
[ 7) I start the washing machine when it is full.]	− 1.520	1.268
[ 8) I choose short programs in the washing machine]	0.413	1.011
[ 9) When buying a new washing machine, I make sure it has a water − saving feature.]	− 1.229	0.477
[ 10) I turn off the tap when shaving or brushing my teeth (for men).]	− 0.253	− 1.485
[ 11) I leave the faucet running during general cleaning]	1.134	0.230
[ 12) I don’t wash the dishes by hand, instead I wash them in the machine to save money.]	− 0.314	− 1.539
[ 13) When I wash the dishes by hand, I wash them in a bowl, not under a running tap ]	0.119	− 1.566
[ 14) I wash the dishes when the machine is full.]	− 0.553	− 1.212
[ 15) When buying a new dishwasher, I make sure it has water − saving features.]	− 0.763	− 0.885
[ 16) I wash fruit and vegetables in the container to save money]	0.407	− 1.299
[ 17) I wipe the balconies clean instead of washing them.]	1.552	1.284
[ 18) I check the plumbing for leaks (e.g. dripping faucet, leaking toilet) I fix the leak immediately.]	− 0.376	− 1.214
[ 19)When buying a water-powered household appliance, I pay attention to its water-saving features.]	− 0.555	− 1.194
[ 20) I report any water leaks I come across to the authorities..]	0.863	− 0.859
[ 21)I make sure to use a broom instead of a hose when cleaning in front of garage doors.]	1.297	0.402
[ 22) I use a bucket rather than a hose when washing the car.]	1.003	− 0.490
[ 23) I avoid watering the garden during the hottest hours of the day.]	0.959	− 0.694

Table 3 below shows the results of water usage consumption patterns (median quartiles).

**Table 3:** Water usage consumption patterns (median quartiles).

Water usage consumption patterns	Never %	Rarely %	Occasionally %	Usually %	Always %	Median	Q1	Q3	Standard deviation
[ 1) I keep my shower time short.]	20.84	9.98	25.72	29.93	13.53	3.05	1.42	3.62	1.33
[ 2) I use low-flow shower heads]	44.07	10.51	15.89	23.04	6.49	2.37	1.57	4.20	1.40
[ 3) When showering and bathing, I do not run the water continuously, I turn the faucet on and off.]	13.05	9.29	20.35	34.96	22.35	3.44	3.13	4.92	1.29
[ 4) I take my bath in a bucket of water.]	32.15	16.85	15.08	14.19	21.73	2.76	1.78	4.77	1.55
[ 5) Instead of flushing, I pour water down the toilet with a bucket]	23.87	13.96	12.84	23.65	25.68	3.13	2.08	5.03	1.53
[ 6) I use the saving siphon with a small and a large tank inside the body.]	44.66	16.38	11.66	18.86	8.44	2.30	1.56	4.12	1.41
[ 7) I start the washing machine when it’s full.]	2.43	5.09	8.85	25.22	58.41	4.32	4.34	5.57	1.00
[ 8) I choose short programs in the washing machine]	16.56	26.17	25.95	23.04	8.28	2.80	2.32	4.27	1.20
[ 9) When buying a new washing machine, I make sure it has a water-saving feature.]	5.38	4.04	12.78	45.96	31.84	3.95	4.06	5.22	1.04
[ 10) I turn off the tap when shaving or brushing my teeth (for men).]	21.07	8.99	13.48	31.46	25.00	3.30	2.44	5.00	1.47
[ 11) I leave the faucet running during general cleaning]	37.30	33.93	14.83	7.87	6.07	2.11	1.67	3.25	1.17
[ 12) I don’t wash the dishes by hand, instead I wash them in the machine to save money.]	18.10	9.05	10.44	35.03	27.38	3.45	2.76	5.09	1.44
[ 13) When I wash the dishes by hand, I wash them in a bowl, not under a running tap ]	23.70	9.24	17.30	37.20	12.56	3.06	2.14	4.67	1.38
[ 14) I wash the dishes when the machine is full.]	12.03	6.37	10.37	37.03	34.20	3.75	3.64	5.27	1.31
[ 15) When buying a new dishwasher, I make sure that it has a water-saving feature]	10.43	5.92	9.72	41.94	31.99	3.79	3.89	5.22	1.25
[ 16) I wash fruit and vegetables in the container to save money]	27.13	10.31	24.22	31.17	7.17	2.81	1.92	4.43	1.32
[ 17)I wipe the balconies clean instead of washing them.]	41.00	25.74	13.44	16.40	3.42	2.15	1.61	3.62	1.22
[ 18)I check the plumbing for leaks (e.g. dripping faucet, leaking toilet) I fix the leak immediately.]	13.20	5.82	15.21	46.98	18.79	3.52	3.39	4.87	1.24
[ 19)When buying a water-powered household appliance, I pay attention to its water saving feature..]	12.95	6.47	12.05	44.42	24.11	3.60	3.50	4.99	1.28
[ 20) I report any water leaks I come across to the authorities.]	32.65	8.16	9.53	26.08	23.58	3.00	1.77	4.95	1.61
[ 21) I make sure to use a broom instead of a hose when cleaning in front of garage doors.]	40.28	24.94	14.42	16.93	3.43	2.18	1.62	3.68	1.23
[ 22) I use a bucket rather than a hose when washing the car.]	46.59	11.99	14.44	23.16	3.82	2.26	1.54	4.09	1.35
[ 23) I avoid watering the garden during the hottest hours of the day.]	46.91	9.83	13.20	24.72	5.34	2.32	1.53	4.21	1.41

Since the data set was normally distributed, Pearson product moment correlation coefficient was used to determine the possible relationship between one water use item and another. Table 4 below shows the results of the Pearson product-moment correlation analysis between water consumption behavior patterns. Pearson correlation coefficient interpretation is given in Table 4 below.

**Table 4:** Pearson correlation analysis results between water consumption behavior patterns.

		1	2	3	4	5	6	7	8	9	10	11	12	13	14	15	16	17	18	19	20	21	22	23
1	Pearson correlation	1																						
	Sig. (2-tailed)																							
	N	451																						
2	Pearson correlation	0.663**	1																					
	Sig. (2-tailed)	0.000																						
	N	447	447																					
3	Pearson correlation	0.064	0.147**	1																				
	Sig. (2-tailed)	0.173	0.002																					
	N	449	445	452																				
4	Pearson correlation	− 0.289**	− 0.320**	0.129**	1																			
	Sig. (2-tailed)	0.000	0.000	0.006																				
	N	448	444	449	451																			
5	Pearson correlation	− 0.194**	− 0.09	− 0.264**	− 0.528**	1																		
	Sig. (2-tailed)	0.000	0.070	0.000	0.000																			
	N	441	437	442	441	444																		
6	Pearson correlation	0.404**	0.464**	− 0.023	0.411**	0.288**	1																	
	Sig. (2-tailed)	0.000	0.000	0.639	0.000	0.000																		
	N	400	397	402	400	394	403																	
7	Pearson correlation	− 0.303**	− 0.241**	0.219**	0.128**	0.128**	− 0.197**	1																
	Sig. (2-tailed)	0.000	0.000	0.000	0.007	0.003	0.000																	
	N	448	444	449	449	442	400	452																
8	Pearson correlation	0.403**	0.253**	− 0.135**	− 0.159**	− 0.224**	0.284**	− 0.172**	1															
	Sig. (2-tailed)	0.000	0.000	0.004	0.001	0.000	0.000	0.000																
	N	443	440	444	443	438	397	444	447															
9	Pearson correlation	− 0.276**	− 0.210**	− 0.023	0.212**	0.223**	− 0.142**	0.413**	− 0.127**	1														
	Sig. (2-tailed)	0.000	0.000	0.000	0.000	0.000	0.005	0.000	0.008															
	N	442	439	443	443	436	396	445	438	446														
10	Pearson correlation	0.216**	0.235**	0.073	− 0.198**	− 0.090	0.214**	0.049	0.234**	0.078	1													
	Sig. (2-tailed)	0.000	0.000	0.171	0.000	0.094	0.000	0.354	0.000	0.145														
	N	352	349	353	352	346	321	353	348	349	356													
11	Pearson correlation	0.292**	0.189**	− 0.123**	− 0.108**	− 0.168**	0.203**	− 0.249**	0.249**	− 0.090	0.134*	1												
	Sig. (2-tailed)	0.000	0.000	0.010	0.023	0.000	0.000	0.000	0.000	0.060	0.013													
	N	441	437	443	441	436	396	442	439	436	347	445												
12	Pearson correlation	0.061	0.108**	0.219**	− 0.152**	− 0.024	0.175**	0.144**	− 0.089	0.174**	0.133*	0.030	1											
	Sig. (2-tailed)	0.206	0.027	0.000	0.002	0.629	0.000	0.003	0.067	0.000	0.014	0.534												
	N	428	424	429	428	422	394	428	424	422	342	422	431											
13	Pearson correlation	0.303**	0.271**	0.191**	− 0.233**	− 0.112**	0.293**	0.050	0.086	0.036	0.227**	0.088	0.227**	1										
	Sig. (2-tailed)	0.000	0.000	0.000	0.000	0.023	0.000	0.310	0.079	0.467	0.000	0.074	0.000											
	N	418	415	419	418	414	385	420	415	415	335	414	418	422										
14	Pearson correlation	0.050	0.000	0.175**	− 0.067	− 0.063	0.005	0.259**	− 0.029	0.279**	0.119*	− 0.033	0.503**	0.322**	1									
	Sig. (2-tailed)	0.305	0.998	0.000	0.171	0.198	0.920	0.000	0.559	0.000	0.030	0.507	0.000	0.000										
	N	420	416	421	420	414	388	421	418	416	334	416	418	411	424									
15	Pearson correlation	− 0.094	− 0.070	0.236**	0.094	0.187**	0.000	0.317**	− 0.083	0.268**	0.124*	− 0.002	0.263**	0.201**	0.251**	1								
	Sig. (2 − tailed)	0.056	0.154	0.000	0.055	0.000	0.993	0.000	0.090	0.000	0.024	0.975	0.000	0.000	0.000									
	N	418	416	420	418	414	386	420	415	415	333	417	415	410	411	422								
16	Pearson correlation	0.495**	0.433**	0.045	0.335**	− 0.198**	0.365**	− 0.127**	0.333**	− 0.140**	0.277**	0.171**	0.097*	0.255**	0.073	− 0.038	1							
	Sig. (2-tailed)	0.000	0.000	0.346	0.000	0.000	0.000	0.007	0.000	0.003	0.000	0.000	0.047	0.000	0.137	0.441								
	N	443	439	444	443	438	396	443	439	437	349	438	423	413	416	416	446							
17	Pearson correlation	0.561**	0.525**	0.062	− 0.198**	− 0.115*	− 0.465**	− 0.212**	0.292**	− 0.209**	0.194**	0.284**	0.111*	0.306**	0.037	− 0.025	0.415**	1						
	Sig. (2 − tailed)	0.000	0.000	0.192	0.000	0.017	0.000	0.000	0.000	0.000	0.000	0.000	0.024	0.000	0.455	0.611	0.000							
	N	436	432	438	436	430	390	437	433	431	342	430	418	410	411	410	431	439						
18	Pearson correlation	0.105*	0.207**	0.251**	− 0.076	0.142**	0.193**	0.102*	− 0.077	0.176**	0.063	− 0.005	0.303**	0.263**	0.381**	0.231**	0.057	0.157**	1					
	Sig. (2-tailed)	0.027	0.000	0.000	0.108	0.003	0.000	0.032	0.109	0.000	0.241	0.915	0.000	0.000	0.000	0.000	0.237	0.001						
	N	444	441	445	445	438	397	445	439	440	349	437	424	416	416	417	439	434	447					
19	Pearson correlation	− 0.115*	− 0.004	0.264**	0.048	0.249**	0.063	0.199**	− 0.177**	0.234**	− 0.030	− 0.115*	0.253**	0.099*	0.292**	0.320**	− 0.001	0.018	0.389**	1				
	Sig. (2-tailed)	0.015	0.938	0.000	0.311	0.000	0.212	0.000	0.000	0.000	0.570	0.016	0.000	0.044	0.000	0.000	0.981	0.704	0.000					
	N	445	441	447	446	440	399	446	441	441	350	440	425	415	418	416	442	434	441	448				
20	Pearson correlation	0.542**	0.571**	0.149**	− 0.580**	− 0.335**	0.543**	− 0.083	0.323**	− 0.116*	0.319**	0.268**	0.238**	0.372**	0.227**	0.074	0.505**	0.485**	0.407**	0.137**	1			
	Sig. (2-tailed)	0.000	0.000	0.002	0.000	0.000	0.000	0.084	0.000	0.015	0.000	0.000	0.000	0.000	0.000	0.132	0.000	0.000	0.000	0.004				
	N	438	436	439	439	432	393	438	435	433	345	431	418	410	413	411	433	428	436	435	441			
21	Pearson correlation	0.539**	0.508**	0.080	− 0.310**	− 0.109*	0.434**	− 0.154**	0.301**	− 0.187**	0.252**	0.261**	0.135**	0.259**	0.056	0.007	0.403**	0.637**	0.227**	0.069	0.548**	1		
	Sig. (2-tailed)	0.000	0.000	0.095	0.000	0.023	0.000	0.001	0.000	0.000	0.000	0.000	0.006	0.000	0.259	0.882	0.000	0.000	0.000	0.151	0.000			
	N	434	431	436	435	429	392	434	432	429	340	429	416	409	410	408	430	427	433	433	428	437		
22	Pearson correlation	0.537**	0.590**	0.087	− 0.442**	− 0.188**	0.532**	− 0.139**	0.293**	− 0.164**	0.273**	0.310**	0.195**	0.308**	0.072	0.073	0.428**	0.550**	0.273**	0.087	0.719**	0.585**	1	
	Sig. (2-tailed)	0.000	0.000	0.097	0.000	0.000	0.000	0.008	0.000	0.002	0.000	0.000	0.000	0.000	0.176	0.178	0.000	0.000	0.000	0.099	0.000	0.000		
	N	364	361	365	364	360	336	364	360	359	344	358	358	351	350	346	361	353	361	362	357	355	367	
23	Pearson correlation	0.539**	0.584**	0.141**	− 0.519**	− 0.237**	0.491**	− 0.139**	0.232**	− 0.122*	0.270**	0.240**	0.221**	0.349**	0.130*	0.027	0.549**	0.556**	0.266**	0.123*	0.807**	0.595**	0.646**	1
	Sig. (2-tailed)	0.000	0.000	0.008	0.000	0.000	0.000	0.009	0.000	0.023	0.000	0.000	0.000	0.000	0.017	0.627	0.000	0.000	0.000	0.021	0.000	0.000	0.000	
	N	353	349	354	354	348	325	353	348	347	298	348	341	335	335	335	353	343	350	351	347	345	305	356

**Correlation significance level is at 0.01.(2-tailed).

*Correlation significance level is at 0.05.(2-tailed).

Pearson correlation coefficient takes values between − 1 and + 1. If the correlation coefficient is − 1, it indicates a perfect relationship in a negative direction. + 1 indicates a perfect relationship in a positive direction. 0 indicates no relationship.

When Table 5 is examined, The highest observed correlation coefficient is 0.807, indicating a strong positive association between 23 (I avoid watering the garden during the hottest hours of the day) and 20 (I report the water leaks I come across to the authorized units). The significance of the correlation coefficient is at 0.01 level. Again, there is a high positive correlation of + 0.719 between 22. (I use a bucket rather than a hose when washing the car) and 20. (I report the water leaks I come across to the authorized units). The significance of the correlation coefficient is at 0.01 level.

**Table 5:** Pearson correlation coefficient interpretation.

r	Value
0.00–0.25	Very weak
0.26–0.49	Weak
0.50–0.69	Middle
0.70–0.89	High
0.90–1.00	Very high

Other several statistically significant correlations are between 23.( I avoid watering the garden during the hottest hours of the day) and 22.( I use a bucket rather than a hose when washing the car) at a rate of + 0.646. The significance of the correlation coefficient is at 0.01 level. There is a + 0.595 correlation between 23.( I avoid watering the garden during the hottest hours of the day) and 21.( I take care to use a broom instead of a hose when cleaning the front of the garage door). The significance of the correlation coefficient is at 0.01 level. There is a + 0.585 correlation between 22.( I use a bucket rather than a hose when washing the car) and 21.( I use a broom instead of a hose when cleaning the front of the garage door). The significance of the correlation coefficient is at 0.01 level.

In addition to the above, there is a + 0,556 correlation between 23.( I avoid watering the garden during the hottest hours of the day) and 17.( I wipe the balconies instead of washing them). The significance of the correlation coefficient is at 0.01 level. There is a + 0.550 correlation between 22.( I use a bucket rather than a hose when washing the car) and 17.( I wipe the balconies instead of washing them). The significance of the correlation coefficient is at the 0.01 level.23.( I avoid watering the garden during the hottest hours of the day) and 16.( I wash fruits and vegetables in a container to save money) are + 0.549. The significance of the correlation coefficient is at 0.01 level. There is a + 0.584 correlation between 23.( I avoid watering the garden during the hottest hours of the day) and 2.( I use low-flow shower heads). The significance of the correlation coefficient is at 0.01 level. There is a + 0.539 correlation between 23.( I avoid watering the garden during the hottest hours of the day) and 1.( I keep my shower time short). The significance of the correlation coefficient is at 0.01 level. There is a + 0.537 correlation between 22.( I use a bucket rather than a hose when washing the car) and 1.( I keep my shower time short). The significance of the correlation coefficient is at 0.01 level.

In addition, there is a + 0.528 correlation between 5.( I pour water into the toilet with a bucket instead of using the flush button) and 4.( I take my bath by filling water in a bucket). The significance of the correlation coefficient is 0.01. There is a + 0.663 correlation between 2nd( I use low-flow shower heads) and 1st( I keep my shower time short). The significance of the correlation coefficient is at 0.01 level.

Several statistically negative significant correlations (an increase in one and a decrease in the other) were identified between 23.( I avoid watering the garden during the hottest hours of the day) and 4.( I take my bath by filling a bucket with water) at a rate of − 0.519. The significance of the correlation coefficient is at 0.01 level. Again, − 0.580 correlation was found between 20.( I report the water leaks I come across to the authorized units) and 4.( I fill my bathroom with water in a bucket). The significance of the correlation coefficient is at 0.01 level.

At the 1% level, there are many significant rank correlations among other water use habits. However, these correlations are weak or very weakly correlated and therefore not significant. Only 64 of the 253 correlations are above 0.30 (in absolute value). Interestingly, however, none of the 57 items showed a relationship between one water use practice and another.

To improve the interpretability of the correlation analysis and avoid item-by-item repetition, water use behaviors were grouped into four main categories: indoor water use, household appliance use, outdoor water use, and civic engagement-related behaviors.

#### Indoor water use behaviors,

Including shower duration, use of low-flow showerheads, turning off taps during personal hygiene, and washing dishes in a bowl, showed moderate positive correlations among themselves. This suggests that households adopting one indoor water-saving practice are more likely to adopt other similar practices, although these relationships do not indicate uniform or systematic behavior across all indoor activities.

#### Household appliance use behaviors,

Such as operating washing machines and dishwashers only when fully loaded and preferring water-efficient appliances, exhibited relatively consistent moderate correlations. These findings indicate that efficiency-oriented appliance use tends to occur as a cluster of related practices, likely reflecting practical or economic considerations rather than broader environmental attitudes.

#### Outdoor water use behaviors,

Including garden watering, car washing, and balcony cleaning practices, showed some of the strongest correlations observed in the analysis. The tendency to avoid watering gardens during peak hours was positively associated with water-saving car washing and cleaning behaviors. Outdoor water use practices show relatively stronger associations than some indoor practices. This may be related to their visibility and discretionary nature.

#### Civic engagement-related behaviors,

Particularly reporting water leaks and promptly repairing household plumbing issues, were positively correlated with several outdoor and indoor conservation practices. This indicates that households engaging in civic-oriented water-saving actions may also be more attentive to specific conservation behaviors. However, these associations remain behavior-specific and should not be interpreted as evidence of comprehensive environmental responsibility.

Overall, the categorized analysis demonstrates that certain water-saving practices tend to be adopted together within thematic domains. Nevertheless, the correlations do not imply a consistent or holistic pattern of water conservation behavior across all categories, and the findings remain limited to associations between specific self-reported practices.

### Trends in water use behavior

**Fig. 1:**
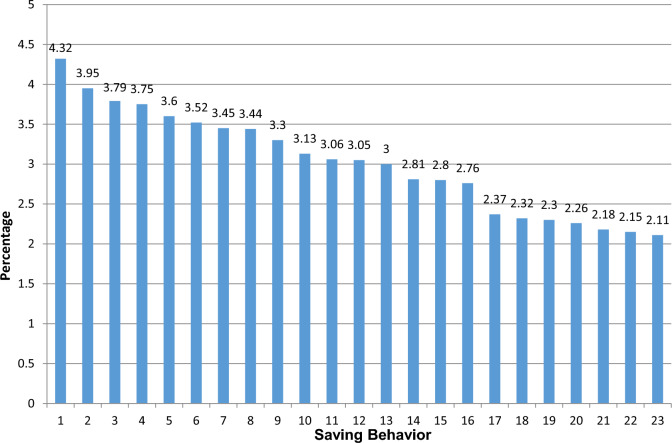
Percentage levels of participants’ mean levels of participation in water use behaviors.

Percentage levels of the subjects’ mean levels of participation in water use behaviors are given in Fig. 1 below.

The highest mean value of the participants’ participation in the behavior was obtained from the statement "I run the washing machine when it is full" (4.32)." When buying a new washing machine, I pay attention to the water saving feature" (3.95). The third most applied item for saving is "I pay attention to water saving features when buying a new dishwasher" with a rate of (3.79). In the participants’ participation in the behavior, the mean of the item "I wash the dishes when the machine is full" was (3.75). In 5th place is the item "I pay attention to the water saving feature when buying a water-powered household appliance" with a rate of (3.60). The items with the lowest mean value of the participants’ participation in the behavior are "I take care to use a broom instead of a hose when cleaning the front of the garage door" with an average of (2.18), "I wipe the balconies instead of washing them" with an average of (2.15), "I leave the tap open during general cleaning" with an average of (2.11). The means and standard deviations of the responses to the 23 questions asked in the questionnaire covering the answers received for water saving are given in Table 6 below.

**Table 6:** Participants’ mean agreement with the statements on water use behaviors.

		Wa	Sd
1	I start the washing machine when it is full	4.32	1.00
2	When buying a new washing machine, I make sure it has a water-saving feature	3.95	1.04
3	When buying a new dishwasher, I make sure it has water-saving features	3.79	1.25
4	I wash the dishes when the machine is full	3.75	1.31
5	When buying a water-powered household appliance, I pay attention to its water saving feature	3.60	1.28
6	I check the plumbing for leaks	3.52	1.24
7	I don’t wash the dishes by hand, instead I wash them in the machine to save money	3.45	1.44
8	When showering and bathing, I do not run the water continuously, I turn the faucet on and off	3.44	1.29
9	I turn off the tap when shaving or brushing my teeth (for men)	3.30	1.47
10	Instead of flushing, I pour water down the toilet with a bucket	3.13	1.53
11	When I wash the dishes by hand, I wash them in a bowl, not under a running tap	3.06	1.38
12	I keep my shower time short.	3.05	1.33
13	I report any water leaks to the authorities	3.00	1.61
14	I wash fruit and vegetables in the container to save money	2.81	1.32
15	I choose short programs in the washing machine	2.80	1.20
16	I take my bath in a bucket of water	2.76	1.55
17	I use low-flow shower heads	2.37	1.40
18	I avoid watering the garden during the hottest hours of the day	2.32	1.41
19	I use a saving siphon with a small and a large tank inside the body	2.30	1,41
20	I use a bucket rather than a hose when washing the car	2.26	1.35
21	I make sure to use a broom instead of a hose when cleaning in front of garage doors	2.18	1.23
22	I wipe the balconies clean instead of washing them	2.15	1.22
23	I leave the faucet running during general cleaning	2.11	1.17

*n = 455 The number and rate of non-respondents are not reflected.

## Conclusions

This study descriptively examines household self-reported water usage and conservation practices in the city center of Isparta. Descriptive statistics and Pearson correlation analysis were used in the study. The research, conducted using survey data and correlation analysis, identified statistically significant relationships among selected water conservation behaviors within the study sample.

The findings of this study indicate that certain water use behaviors tend to be associated with one another within the study sample. Several statistically significant correlations were identified; however, these relationships should be interpreted as associations rather than evidence of causal or systematic behavior patterns.

The results do not suggest that households consistently adopt all water-saving practices, nor do they imply a uniform level of awareness or environmental responsibility. Instead, the findings reflect behavior-specific tendencies observed in self-reported data.

The survey identified 28 statistically significant correlations among the items examined. The existence of a strong correlation between two specific practices does not indicate that households systematically adopt all water-saving behaviors. The analysis only provides insight into the relationships between selected practices and does not constitute a comprehensive assessment of overall water-saving behavior.

The study does not claim that households have the same high level of awareness or similar attitudes regarding water conservation. The findings are limited to the statistical relationships observed between specific self-reported behaviors within the study period and sample.

One important limitation of this study is that the data were collected between 2009 and 2013. Therefore, the findings reflect the behavioral patterns and conditions specific to that period. Changes in technology, water pricing policies, infrastructure, and public awareness over time may influence current water use behaviors differently.

However, the dataset still provides valuable insights into the relationships between household water use practices. The results should be interpreted as exploratory and context-specific rather than representative of current conditions. Future studies using updated datasets and longitudinal approaches are recommended to validate and extend these findings.

Despite the temporal limitation of the dataset, the study contributes to the literature by providing an empirical baseline for understanding the structure of water use behaviors and their interrelationships in a medium-sized urban context.

In addition, the dataset used in this study can serve as a benchmark for future research, enabling comparative analyses of changes in household water use behaviors over time.

## Data Availability

The datasets used and/or analysed during the current study are available from the corresponding author on reasonable request.

## References

[1] Chen, X., Li, J. & Chen, C. The role of social capital in water conservation: Evidence from China. *Environ. Sci. Technol.* **56**(16), 10664–10673 (2022).

[2] Larson, J. & Tveit, M. Urban water demand management: A review of recent literature. journal of water resources planning and management. **148**(4) (2022).

[3] Stringer, L. C. et al. Climate change ımpacts on water security in global drylands. *One Earth* **4**(6), 851–864 (2021).

[4] Basu, M., DasGupta, R., Hashimoto, S. & Hoshino, S. A multi-actor and bottom-up perspective on attaining rural water security: Qualitative evidence from India. *Environ. Dev. Sustain.* **23**(2), 1461–1484 (2021).

[5] Casas, M. V., March, H. & Sauri, D. Examining the reduction in potable water consumption by households in Catalonia (Spain): Structural and contingent factors. *Appl. Geogr.* **87**, 234–244 (2017).

[6] Almulhim, I. A. & Abubakar, I. R. A segmentation approach to understanding water consumption behavioral patterns among households in Saudi Arabia for a sustainable future. *Resourc., Environ. Sustain.* **15**, 100144 (2024).

[7] Grespan, A., Garcia, L., Brikalski, M. P., Henning, E. & Kalbusch, A. Assessment of water consumption in households using statistical analysis and regression trees. *Sustain. Cities Soc* **87**, 104186 (2022).

[8] Koop, S. H. A., Van Dorssen, A. J. & Brouwer, S. Enhancing domestic water conservation behaviour: A review of empirical studies on influencing tactics. *J. Environ. Manag.* **247**, 867–876 (2019). 10.1016/j.jenvman.2019.06.126 31376785

[9] Araya, F., Osman, K. & Faust, K. M. Perceptions versus reality: Assessing residential water conservation efforts in the household. *Resour. Conserv. Recycl.* **162**, 105020 (2020).

[10] Singha, B., Karmaker, S. C. & Eljamal, O. Quantifying the direct and indirect effect of socio-psychological and behavioral factors on residential water conservation behavior and consumption in Japan. *Resour. Conserv. Recycl.* **190**, 106816 (2023).

[11] Stoker, P. & Rothfeder, R. Drivers of urban water use. *Sustain. Cities Soc.* **12**, 1–8 (2014).

[12] Akgül, A. & Çevik, O. *İstatistiksel analiz teknikleri* (Emek ofset, 2003).

[13] Nakip, M. pazarlama araştırmaları teknikler ve (spss destekli)uygulamalar (Seçkin yayıncılık, Ankara 2003).

[14] Yükselen, C. Pazarlama araştırmaları (Detay yayıncılık, Ankara, 2000).

[15] Kartal, M. Bilimsel araştırmalarda hipotez testleri (Nobel yayın dağıtım, Ankara, 2006).

[16] Gürsakal, N. Bilgisayar uygulamalı istatistik II. Marmara kitabevi. Bursa. 517s (1998)

[17] Kayaalp, G.T. ve Çankaya, S. İstatistik. Ç.Ü. ziraat fakültesi genel yayın no:258. Ders kitapları yayın no: A-84. Adana. 122s (2003)

[18] Tabachnick, B.G., Fidell, L. S. Using multivariate statistics, 6th edn, 1018 (Courier Companies Inc, Northridge, 2013)

[19] George, D., Mallery, F. SPSS For windows step by step: A simple guide and reference fourth edition (11.0 Update) 386 (Allyn and Bacon, Boston, 2010).

